# Hydroxychloroquine Protects against Cardiac Ischaemia/Reperfusion Injury *In Vivo* via Enhancement of ERK1/2 Phosphorylation

**DOI:** 10.1371/journal.pone.0143771

**Published:** 2015-12-04

**Authors:** Lauren Bourke, James McCormick, Valerie Taylor, Charis Pericleous, Benoit Blanchet, Nathalie Costedoat-Chalumeau, Daniel Stuckey, Mark F. Lythgoe, Anastasis Stephanou, Yiannis Ioannou

**Affiliations:** 1 Centre for Rheumatology, Division of Medicine University College London, Rayne Institute, London, United Kingdom; 2 Arthritis Research UK Centre for Adolescent Rheumatology, University College London, London, United Kingdom; 3 Biochemistry Research Group, Clinical and Molecular Genetics Unit, Institute of Child Health & Great Ormond Street Hospital, University College London, London, United Kingdom; 4 UCL Centre for Advanced Biomedical Imaging, Division of Medicine, London, United Kingdom; 5 Assistance Publique Hôpitaux de Paris, Hôpital Cochin, Unité Fonctionnelle de Pharmacocinétique et Pharmacochimie, Paris, France; 6 Université René Descartes; Centre de référence maladies auto-immunes et systémiques rares, Service de Médecine Interne, Pôle médecine, Hôpital Cochin, AP-HP, Paris, France; 7 Medical and Molecular Biology Unit, University College London, London, United Kingdom; Indiana University School of Medicine, UNITED STATES

## Abstract

An increasing number of investigations including human studies demonstrate that pharmacological ischaemic preconditioning is a viable way to protect the heart from myocardial ischaemia/reperfusion (I/R) injury. This study investigated the role of hydroxychloroquine (HCQ) in the heart during I/R injury. *In vitro* and *in vivo* models of myocardial I/R injury were used to assess the effects of HCQ. It was found that HCQ was protective in neonatal rat cardiomyocytes through inhibition of apoptosis, measured by TUNEL and cleaved caspase-3. This protection *in vitro* was mediated through enhancement of ERK1/2 phosphorylation mediated by HCQ in a dose-dependent fashion. A decrease in infarct size was observed in an *in vivo* model of myocardial I/R injury in HCQ treated animals and furthermore this protection was blocked in the presence of the ERK1/2 inhibitor U0126. For the first time, we have shown that HCQ promotes a preconditioning like protection in an *in vivo* simulated rat myocardial I/R injury model. Moreover, it was shown that HCQ is protective via enhanced phosphorylation of the pro-survival kinase ERK1/2.

## Introduction

An increasing number of investigations have demonstrated that pharmacological preconditioning induces a cardioprotective effect against I/R injury, with examples including sildenafil and cyclosporine A [1,2]. Preconditioning was originally described in 1986 by *Murry et al* [3] who found that four cycles of 5 minute left circumflex coronary artery occlusions, before a 40 minute occlusion, reduced MI size by 75%. Since then many studies have confirmed this in both the heart and other organs and there are currently a number of ongoing clinical trials to explore the therapeutic potential of this effect [4,5]. This includes protecting a patient’s heart prior to surgery by preconditioning via mechanisms such as remote ischaemic preconditioning (RIPC), which is currently being explored in the ERICCA trial in patients undergoing coronary artery bypass graft (CABG) ± valve surgery [5].

The mitogen activated protein (MAP) kinase family are serine-threonine kinases which play a role in I/R injury [6,7]. The three major family members that have been extensively studied in the heart are c-Jun N-terminal kinases (JNK1 and JNK2), p38 kinases (of which p38α and p38β isoforms are found in the heart) and extracellular signal-regulated kinases (ERK1 and ERK2) [8]. The first two are known to enhance apoptosis but the latter has been shown to mediate protection when its phosphorylation state is increased, thus is cardioprotective [6]. Inhibition of ERK1/2 phosphorylation during I/R injury has been shown to enhance apoptosis [9,10]. ERK1/2 along with another pro-survival kinase Akt (protein kinase B) constitutes the reperfusion injury salvage kinase (RISK) pathway [11]. The RISK pathway has been identified as the pathway that is up-regulated via pre-conditioning thus providing protection. It therefore may be possible to increase protection by enhancing these pathways, making them an appealing therapeutic target [10,12].

An unconventional function of the autophagy ATG proteins in the regulation of ERK1/2 phosphorylation has recently been shown [13]. Deleting Atg7 or Atg5 or blocking LC3 lipidation was shown to decrease ERK1/2 phosphorylation and conversely, increasing LC3-II (light chain 3) availability increased ERK1/2 phosphorylation. Therefore regulation of LC3 lipidation is a potential target to regulate levels of the therapeutic kinase ERK1/2. The drug hydroxychloroquine (HCQ), originally an anti-malarial, is now used to treat autoimmune diseases such as systemic lupus erythematosus (SLE) and rheumatoid arthritis [14,15]. HCQ inhibits autophagy by altering the pH of the lysosome, therefore preventing the breakdown of autophagosomes [16]. These intact autophagosomes have various membrane proteins attached, such as the autophagy marker LC3-II, resulting in an increase and persistence in their expression [17]. The identification of this autophagy mediated mechanism has led to HCQ being re-purposed for use in cancer [18], due to cancer cells enhancing autophagy as a mechanism to resist death [17,19]. Given that LC3-II enhancement is linked to increases in phosphorylation of the pro-survival kinase ERK1/2 [13] and HCQ causes an accumulation of intracellular autophagosomes our study aimed to explore whether HCQ could enhance ERK1/2 phosphorylation, consequently leading to protection of the heart during I/R injury as a pharmacological pre-conditioner.

## Results

### HCQ reduces cell death in I/R injury *in vitro*

**Fig 1:**
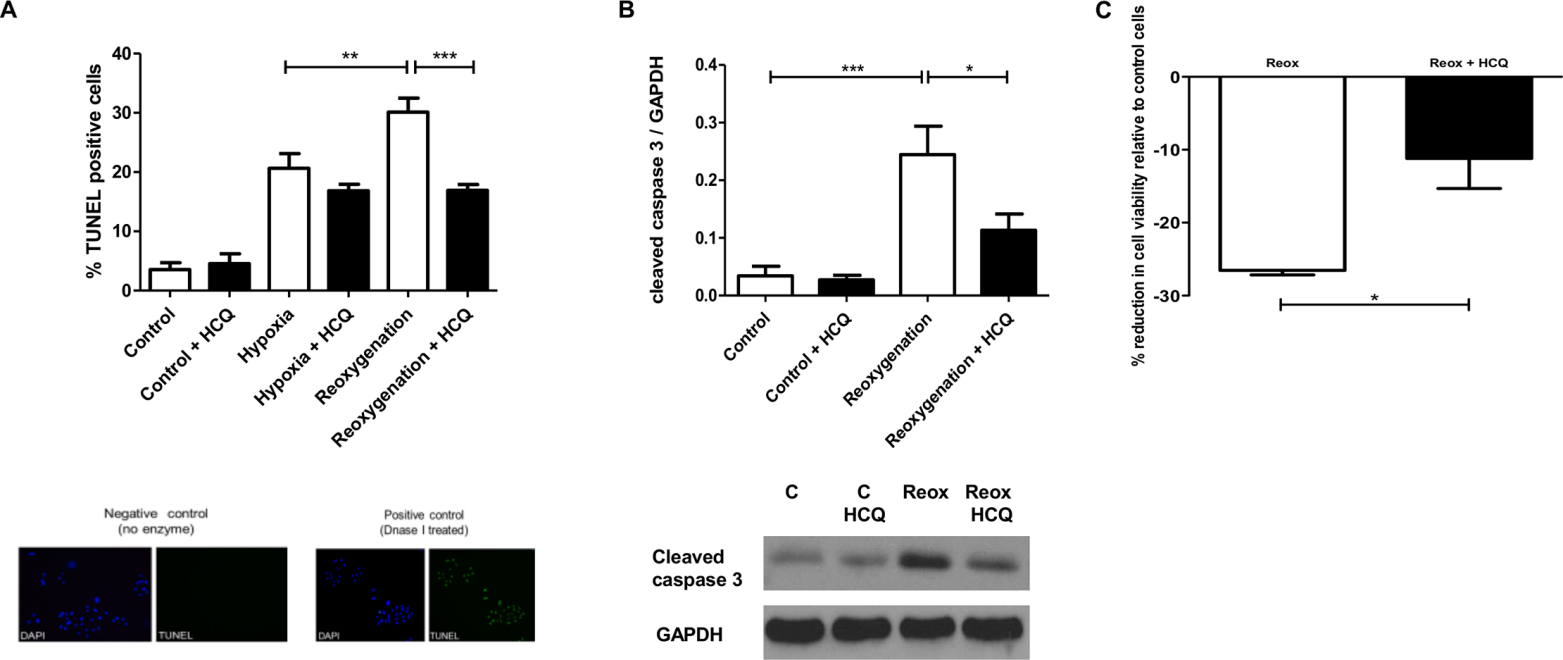
HCQ reduces apoptosis and total cell death in neonatal rat cardiomyocytes in simulated I/R injury. Cells were pre-treated overnight with 2000 ng/mL HCQ. The following day cells were exposed to simulated I/R injury (4h hypoxia + 16h (A) or 2h (B) reoxygenation) in the presence of HCQ and the percentage of apoptotic cells (A + B) and total cell death (C) was assessed using TUNEL (A), western blotting (B) and cell viability assay (C). Graphs show ±SEM of quantitative analysis from nine (A), four (B) and three (C) independent experiments. Statistical analysis determined by 1 way ANOVA using post-hoc Tukey to compare all columns (* p<0.05, ** p<0.005, *** p<0.0005) (A and B) and unpaired t-test (p = 0.0213) (C).

An *in vitro* simulated model of cardiac I/R injury was used, whereby neonatal rat cardiomyocytes were isolated and treated with 2000 ng/ml HCQ, which approximates to the physiological concentrations achieved in patients [20]. Cells exposed to hypoxia alone had 20.65% (±SD 7.38) TUNEL positivity and when exposed to reoxygenation for 16 hours this is enhanced to 30.13% (±SD 7.05, p<0.005) (Fig 1A). However, when cells are pre-incubated with HCQ. this enhancement of TUNEL positivity during the reoxygenation stage is completely abrogated back down to below that observed in cells exposed to hypoxia alone (16.93% (±SD 3.00, p<0.0005)). When probing for cleaved capsase-3, another downstream marker of apoptosis, HCQ showed the same protective effect during the simulated reperfusion stage. Cleaved caspase-3 was increased during reoxygenation when compared to cells kept in optimal conditions (0.24 relative to GAPDH (±SD 0.09) vs 0.03 relative to GAPDH (±SD 0.03)(p<0.0005)). In the presence of HCQ, this increase in cleaved caspase-3 was significantly reduced by 54.16% (0.11 relative to GAPDH (±SD 0.05, p<0.05) (Fig 1B). A colorimetric cell proliferation assay confirmed that HCQ caused a reduction in total cell death in cells exposed to simulated I/R injury of 57.89% (±SD 7.14, p = 0.0213) (Fig 1C). Additional experiments showed that the protective effects of HCQ were most prominent when cells were pre-incubated with HCQ prior to simulated I/R and when it remained present throughout the experiment. This was measured by assessing levels of cleaved caspase-3 (S1A Fig).

### HCQ enhances phosphorylation of the pro-survival kinase ERK1/2 *in vitro*

**Fig 2:**
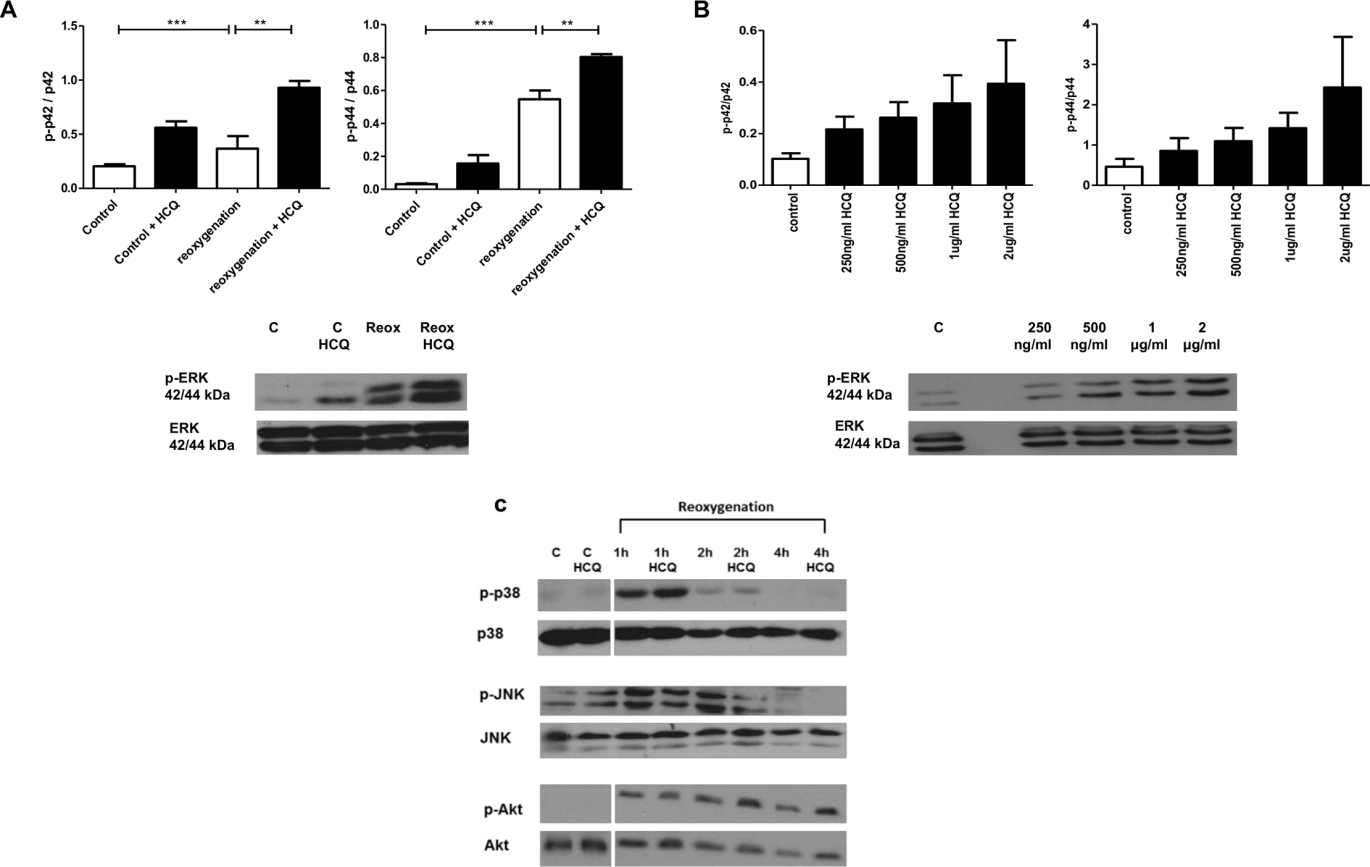
HCQ increases the pro-survival kinase ERK1/2 phosphorylation in simulated I/R injury in neonatal rat cardiomyocytes and is dose-dependent. Western blot analysis of cell lysates from neonatal rat cardiomyocytes pre-treated overnight with 2000 ng/mL (A) or varying concentrations (250 ng/mL to 2000 ng/mL) of HCQ (B) and the following day exposed to simulated I/R injury (4h hypoxia + 2h reoxygenation) with HCQ present throughout. HCQ increases ERK phosphorylation for both p44 and p42 isoforms. No significant difference is observed in phosphorylation of JNK and Akt after 4h hypoxia + 1h, 2h and 4h reoxygenation (C). Additionally, no difference was observed in phosphorylation of ERK5 in cells pre-treated with HCQ overnight and the following day exposed to simulated I/R injury (4h hypoxia + 4h reoxygenation). Graph shows ±SEM of quantitative analysis from five (A), three (B) and three (C) independent experiments. Statistical analysis determined by 1 way ANOVA using post-hoc Tukey to compare all columns (** p<0.005, ***p<0.0005).

Enhancement of ERK1/2 phosphorylation was observed in the presence of HCQ at 2 hours post reoxygenation for both p44 and p42 isoforms (Fig 2A). In cells exposed to reoxygenation phosphorylated p44 ERK had a mean of 0.55 (±SD 0.12) versus 0.8 (±SD 0.04)(p<0.0005) in HCQ treated cells (units relative to total p44). For phosphorylated p42 ERK, reoxygenation alone had a level of 0.37 (±SD 0.23) versus 0.93 (±SD 0.12)(p<0.0005)(units relative to total p42) in HCQ treated cells. This enhancement of ERK1/2 phosphorylation by HCQ was also shown to be dose dependent (Fig 2B). However, HCQ did not significantly affect levels of Akt, JNK, or p38 MAPK (Fig 2C). ERK1/2 phosphorylation was increased most when cells were pre-treated with HCQ and then incubated with the drug throughout the whole experiment (S1B Fig).

**Fig 3:**
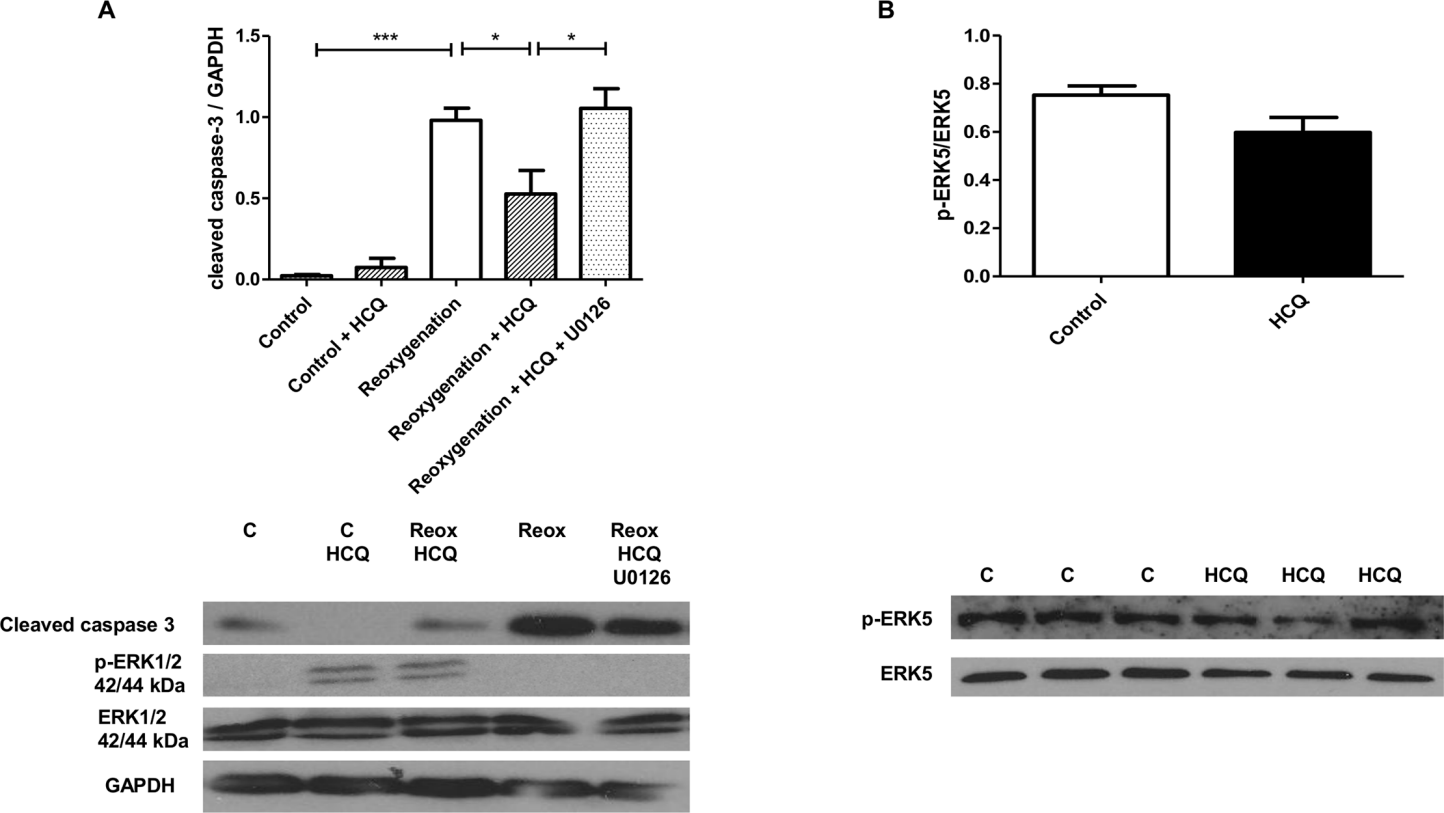
U0126 reverses the cardioprotective effect of HCQ in simulated I/R injury in neonatal rat cardiomyocytes. Western blot analysis of cell lysates from neonatal rat cardiomyocytes pre-treated overnight with 2000 ng/mL HCQ and the following day incubated with 10 μM U0126 for 2 hours followed by simulated I/R injury (4h hypoxia + 2h reoxygenation). Immunoblots were probed for cleaved caspase-3, ERK1/2 phosphorylation (A) and ERK5 phosphorylation (B). Graphs show mean ±SEM of quantitative analysis from four independent experiments. Statistical analysis determined by 1 way ANOVA using post-hoc Tukey to compare all columns (*p<0.05, *** p<0.0005)(a).

When cells were co-incubated with an ERK1/2 specific inhibitor (U0126), the protection mediated via HCQ was completely abrogated (Fig 3A). In the presence of HCQ alone, levels of cleaved caspase 3 were decreased compared to untreated cells (control- 0.98 (±SD 0.21) vs HCQ treated- 0.53 (±SD 0.25)(p<0.05) relative to GAPDH). However, when the inhibitor U0126 and HCQ were added together levels were significantly increased to that seen in control cells, therefore blocking protection (control- 0.98 (±SD 0.21) vs U0126 + HCQ treated- 1.06 (±SD 0.21). Additionally, the kinase ERK5 was also probed for, however no differences in ERK5 phosphorylation were observed suggesting that it has no role in the cardioprotective effect of HCQ (Fig 3B).

### HCQ inhibits downstream pathways of ERK1/2 phosphorylation *in vitro*

**Fig 4:**
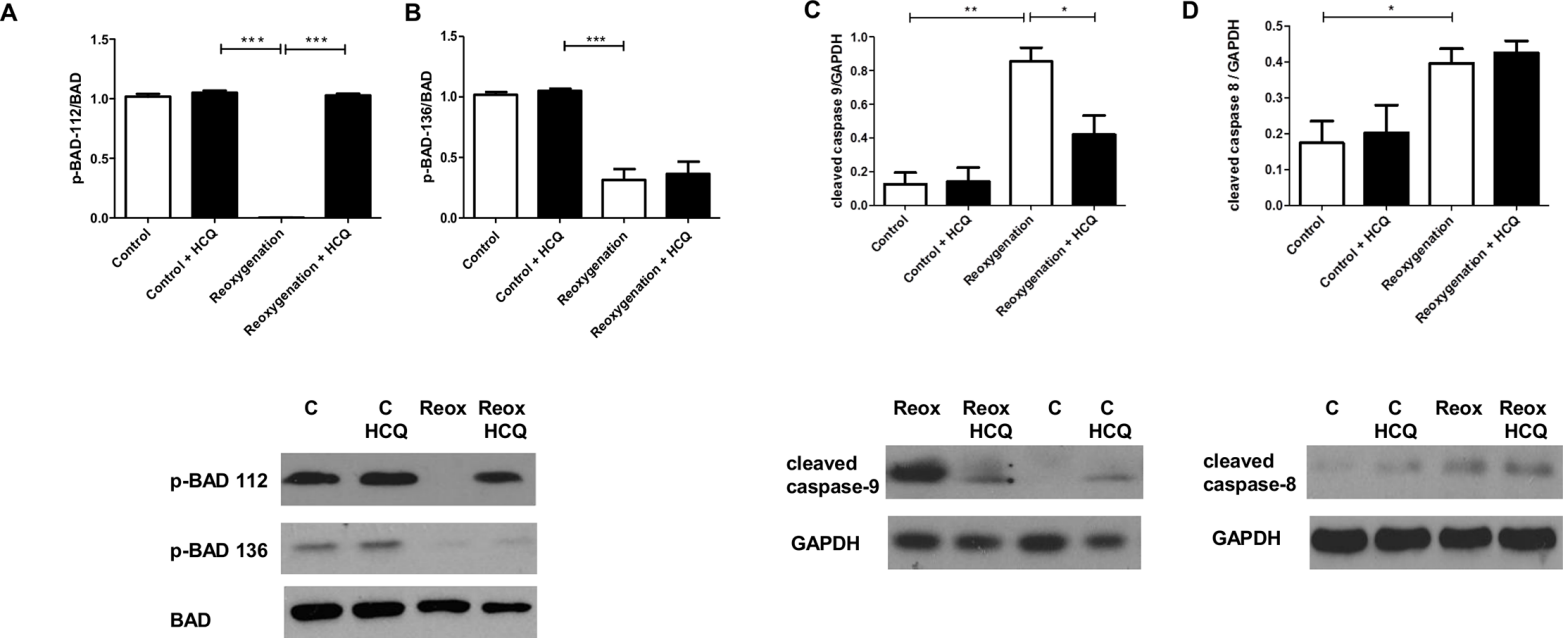
HCQ blocks ERK-dependent dephosphorylating of BAD and reduces cleaved caspase 9 in neonatal rat cardiomyocytes in simulated I/R injury. Western blot analysis of cell lysates from neonatal rat cardiomyocytes pre-treated overnight with 2000 ng/mL HCQ and the following day exposed to simulated I/R injury (4h hypoxia + 2h reoxygenation). BAD dephosphorylating at serine 112 (A) and serine 136 (B) and levels of cleaved caspase-8 and caspase-9 were assessed using western blot. Graphs show mean ±SEM of quantitative analysis from three independent experiments. Statistical analysis determined by 1 way ANOVA using post-hoc Tukey to compare all columns (*p<0.05, ** p<0.005, *** p<0.0005).

The effect of HCQ to pathways downstream of increased ERK1/2 phosphorylation was next explored. It has been shown that phosphorylation of the serine 112 residue of the pro-apoptotic protein BAD is regulated by ERK1/2, whereas phosphorylation of serine 136 of BAD is regulated by Akt [21]. HCQ was shown to block de-phosphorylation of serine 112 (Fig 4A) but not the Akt regulated serine 136 residue (Fig 4B). This further supported our findings that HCQ protection was mediated via modulation of ERK1/2.

Upstream to the execution caspase (caspase-3) being cleaved, there are two distinct pathways; the extrinsic and intrinsic pathways, which are regulated by cleavage of caspase-8 and caspase-9 respectively [22]. BAD has been shown to be involved in the regulation of the intrinsic pathway and subsequently caspase-9 cleavage [21]. Our studies demonstrated that HCQ inhibits caspase-9 cleavage during simulated I/R injury *in vitro* (reoxygenation alone- 0.79 (±SD0.23) vs HCQ treated- 0.44 (±SD0.27)(p0.05) relative to GAPDH) (Fig 4C). However, there was no change in levels of cleaved caspase-8, which occurs independently of BAD activation (Fig 4D). This further confirmed an ERK dependent mechanism whereby BAD activation is blocked by an increase in ERK phosphorylation, leading to a subsequent reduction in cleaved caspase-9.

### HCQ reduces infarct size in an *in vivo* I/R injury model

**Fig 5:**
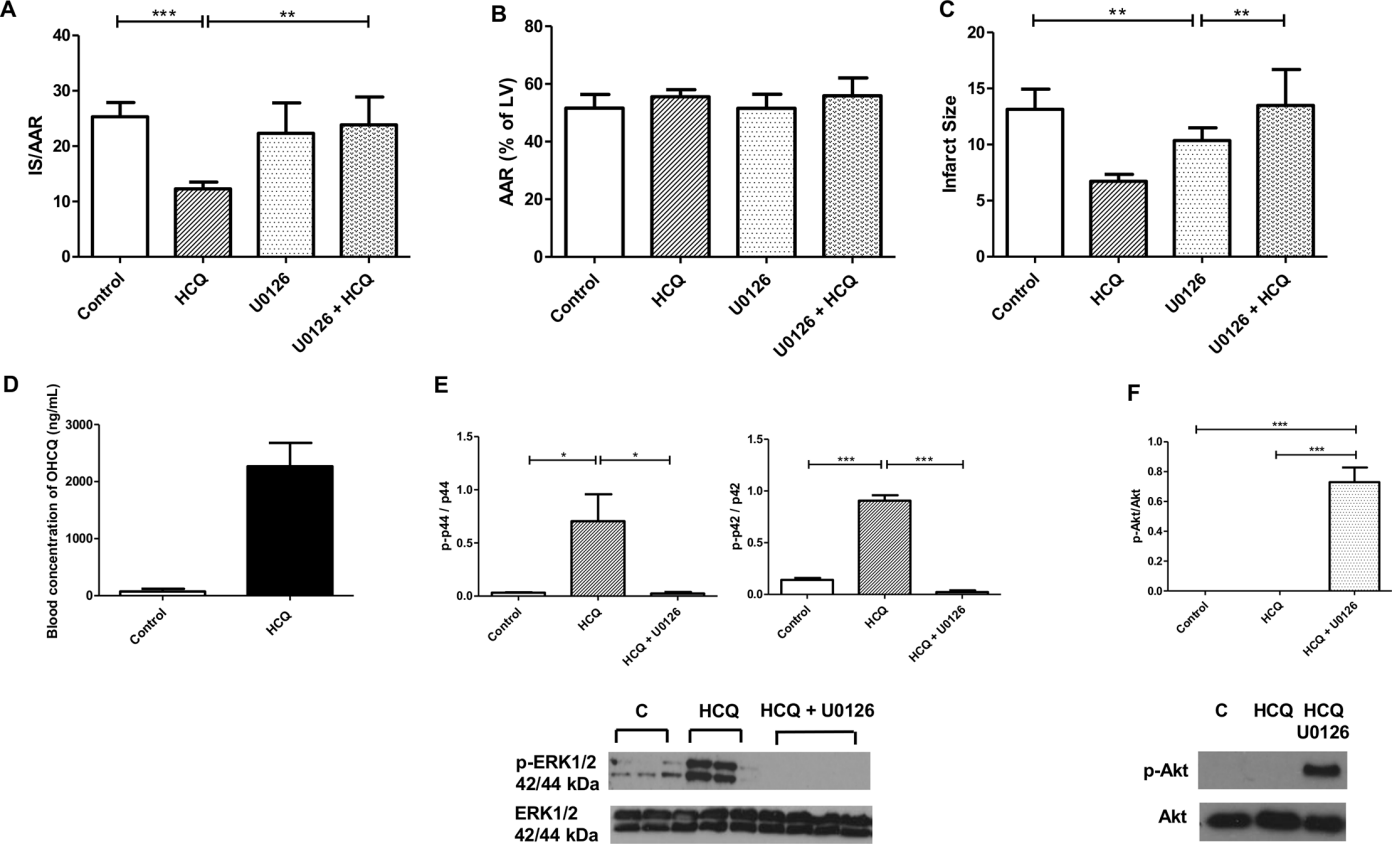
Infarct size is reduced in rats pre-treated with HCQ and then subjected to cardiac I/R injury in vivo and is ERK1/2 dependent. Rats were dosed by gavage with 200 mg/ml HCQ for three days prior to surgery (1h occlusion of LAD artery + 24h reperfusion) and when sacrificed hearts were stained with Evans blue and TTC to assess infarct size (IS) (B) and area at risk (AAR) (C). Graphs A, B and C show mean ±SEM of quantitative analysis from ten control, six U0126 treated, eleven HCQ treated and five HCQ + U0126 treated rats. Blood levels of HCQ were detected using HPLC (D). Rats were treated with 1 mg U0126, 30 minutes prior to surgery to inhibit ERK1/2 phosphorylation. Graphs e and f show mean ±SEM of quantitative analysis from four control, four HCQ treated and five HCQ + U0126 treated rats. Statistical analysis determined by unpaired t-test (*** p = 0.0002, ** p = 0.0077)(A), (** p = 0.0022, ** p = 0.0097)(C) and one way ANOVA with Tukey post hoc analysis (* p<0.5, *** p<0.0005)(E+F).

Oral administration (via gavage) of HCQ for three days prior to I/R injury caused a significant decrease in cardiac infarct size in rats 24 hours after I/R injury, with a reduction of 47%. Control rats infarct size expressed as IS/AAR (infarct size relative to area at risk) was 25.32% (±SD8.1, n = 10) compared to a decrease in the presence of HCQ to 12.28% (±SD5.8, n = 11) (Fig 5A). Furthermore, when rats were treated via intraperitoneal injection with the ERK1/2 inhibitor U0126 prior to surgery, protection by HCQ was abrogated (HCQ treated- 12.28% (±SD5.8)(n = 11) vs HCQ + U0126 treated- 23.86% (±SD11.23)(n = 5). Levels of HCQ achieved in rat blood were 1895 ng/mL (±SD436.9) (Fig 5D) thus reflecting levels that are observed in patients who take HCQ of between 1,000–2,000 ng/mL [20]. Western blot analysis confirmed that pre-administration of U0126 inhibited ERK1/2 phosphorylation in the rat heart (Fig 5E). Rats treated with ERK1/2 inhibitor alone had an infarct size similar to that seen in control rats, which was unexpected as it was predicted that inhibiting ERK1/2 would enhance infarct size. However, when hearts were probed for phosphorylation of the pro-survival kinase Akt a significant increase was observed in the presence of U0126 (Fig 5F).

## Discussion

This report describes a novel protective mechanism for HCQ in a model of acute cardiac I/R injury. Additionally, we have dissected the pathways through which this protection is mediated utilising established *in vitro* and *in vivo* models of cardiac I/R injury. HCQ inhibited cardiomyocyte apoptosis and total cell death in an *in vitro* simulated I/R injury model, and reduced the infarct size in an *in vivo* rat model of I/R injury. The protocol was designed to achieve therapeutic levels of HCQ that are seen in patients treated with this drug [20]. Additional *in vitro* experiments were performed to determine when HCQ had to be administered for maximal cardioprotection. It was observed that protection, as measured by a reduction in cleaved caspase-3, was greatest when cells were incubated with HCQ prior to, during and after simulated I/R injury (S1A Fig). These findings of HCQ being protective in cardiac I/R injury are supported by previous studies that have shown a protective role for chloroquine in other organ systems such as renal [23] and liver I/R injury [24].

It remains unclear as to the contribution of apoptosis to total infarct size, with some studies suggesting it representing only a small percentage of cellular death [25] but others suggesting the use of caspase inhibitors attenuates the damage by I/R injury [26]. However, given that caspase activity has also been shown to also contribute to necrotic cell death [27], it should not be discarded that a proportion of the reduction in infarct size observed could be independent of apoptosis inhibition.

As well as identifying a cardioprotective mechanism of HCQ, we went on to classify a specific mechanism of action through which the drug elucidates its protection. Our data showed a significant increase in ERK1/2 phosphorylation in cells treated with HCQ compared to control cells; this was enhanced further upon exposure to I/R injury both *in vitro* (Fig 2A) and *in vivo* (Fig 5E) as well as being dose-dependent (Fig 2B). It has been previously shown that other drugs such as sildenafil have the same effect, whereby they mimic the RISK pathway in order to prime cells for protection should a myocardial infarction (MI) occur [12]. We went on to show both *in vitro* (Fig 3) and *in vivo* (Fig 5A) that the ERK1/2 inhibitor U0126 blocked this protective mechanism, demonstrating that HCQ protection is ERK1/2 dependent. It should be noted however, that U0126 has been shown to be a weak inhibitor of other signalling pathway proteins [28]. In our study it was observed that U0126 did not have an inhibitory effect on JNK (Fig 2C) or ERK5 (Fig 3B) phosphorylation. Another pathway U0126 has been shown to interact with is the mTOR/autophagy pathway [10,29], given that HCQ is known to inhibit autophagy, subsequent experiments to dissect out the specific mechanism through which U0126 is blocking the protective effects of HCQ should be explored.

Interestingly, in the *in vivo* model of I/R injury the presence of U0126 restored the infarct size to that seen in untreated rats, but did not make it more severe as was expected. We believe that this is due to a cross-talk between ERK1/2 and Akt resulting in Akt compensating for the inhibition of the pro-survival kinase ERK1/2. Our observation that the presence of the U0126 ERK inhibitor enhanced phosphorylation of the pro-survival kinase Akt, supports this (Fig 5F). An increase in Akt phosphorylation to compensate for a reduction in ERK1/2 phosphorylation has previously been reported by others, who suggest that mediators such as p70s6k or BAD, which are both known to interact with Akt and ERK1/2, are responsible for preventing an increase in infarct size [30,31].

Studies have shown that the pro-apoptotic protein BAD requires de-phosphorylation specifically at serine 112 to become activated [32]. BAD has two main serine residues which require de-phosphorylation and are controlled independently of one another, serine 112 by ERK1/2 and serine 136 by Akt [33]. Therefore as observed in our study, increases in ERK1/2 phosphorylation blocked de-phosphorylation of serine 112 and therefore inactivated it (Fig 4A). Further downstream pathways were also investigated and a reduction in caspase 9 cleavage was seen, which has previously been shown to be regulated by BAD (Fig 4C).

Previous studies have suggested that HCQ enhances endothelial ERK5 phosphorylation, therefore exerting vasoprotective properties [34]. However, we found that in neonatal rat cardiomyocytes there was no effect on ERK5 status in the presence of HCQ (Fig 3B), suggesting it does not play a role in the protective effects observed. This protective role of HCQ is supported by a previous study which showed that low doses of HCQ resulted in a reduction in apoptosis in the peri-infarct myocardium [35]. However, our study is the first to associate enhancement of a pro-survival kinase, namely ERK1/2 and importantly a reduction in infarct size. Furthermore, we provide evidence that when ERK1/2 phosphorylation is inhibited, this protection is blocked.

HCQ inhibits autophagy by preventing acidification of the lysosome, making it unable to attach to and breakdown autophagosomes [17]. This leads to accumulation of autophagosomes with markers such as LC3-II assembled on their membrane. HCQ has been explored as a potential therapeutic for cancer due to its known role in inhibiting autophagy, combined with evidence that suggests cancer cells enhance autophagy as a way of resisting death [13,19]. Given that LC3-II has been identified as a scaffold upon which ERK1/2 phosphorylation may occur [13], we hypothesised a protective mechanism through which this LC3-II accumulation may result in increased ERK1/2 phosphorylation and hence protection in cardiac I/R injury. The data presented in this study suggests that autophagy regulation is the likely upstream mechanism of HCQ that results in enhanced ERK1/2 phosphorylation. This is supported by the observation that LC3-II accumulation increases in the presence of HCQ, but this effect is blocked with the autophagy inhibitor 3-Methyladenine (3-MA) which prevents autophagosome formation and therefore LC3-II accumulation (S2A Fig). Furthemore, ERK1/2 phosphorylation is enhanced in the presence of HCQ (S2B Fig), resulting in a reduction in cleaved caspase-3 (S2C Fig), but in the presence of 3-MA this protective effect is removed. These experiments therefore underpin future mechanistic studies targeting aspects of autophagy regulation in order to further dissect the pathways through which HCQ is cardioprotective and mediates enhanced phosphorylation of ERK1/2.

It should be noted that a previous study by Ma et al has suggested HCQ may contribute to cardiomyocyte death due to blocking autophagy, resulting in impaired autophagosome clearance [36]. Whilst Ma et al suggest that the blockade of autophagosome clearance results in enhanced cardiomyocyte cell death I/R injury, the contribution of autophagy to I/R injury remains controversial [37]. In contrast, we have specifically assessed the role of HCQ in cell death via apoptosis *in vitro* and furthermore total cell death (measured by infarct size) *in vivo*, an effect we have identified as being through well-defined pro-survival mechanisms. The longer-term effects of HCQ on myocardial viability post-MI now require elucidation to ascertain if this protective effect is sustained and further support the translational potential of the findings reported here.

A limitation of our study is that the *in vivo* testing of HCQ mediated protection was conducted in rodent models of I/R injury. Previous studies have shown that drugs such as sildenafil, adenosine agonists and cyclosporine show protection in rodents, however translation of these findings to the clinical setting has been disappointing [38]. The reason for the poor performance of conditioning mimetics in the clinic, despite promising animal results has been previously discussed [39,40]. Issues include the influence of drugs previously administered to patients such as statins and platelet inhibitors may result in the heart already being conditioned, as well as co-morbidities potentially resulting in blunt protection from conditioning. At present, the success rate of lead pharmacological candidates targeting pathways relevant to I/R injury, progressing through to market has been disappointing. A major obstacle relates to adverse side effects observed with pharmacological modulators; however HCQ has a favourable safety profile which has been confirmed due to its routine use in rheumatology for the last 50 years [41]. Evidence of an enhanced pre-conditioning like effect in humans would first need to be demonstrated before it can be proposed that the findings of this study could support HCQ having a re-purposed role in I/R injury cardioprotection. To our knowledge, this is the first study demonstrating a preconditioning-like cardioprotective effect of HCQ against I/R injury in an *in vitro* and subsequently *in vivo* rat model. Additionally, we have shown that inhibition of ERK1/2 phosphorylation blocked HCQ-mediated protection, thereby identifying a mechanism of action. The overall safety of HCQ use is well established with long term administration showing a favourable safety profile, thereby enhancing the potential for clinical studies [41]. Given that HCQ has also been shown to have antithrombotic properties [42] and a favourable effect upon lipid profiles [43], its potential benefits described in this study underpin the rationale to investigate this drugs use for a re-purposed role in cardiovascular disease however first evidence of HCQ having a similar effect in humans needs to be demonstrated before such clinical studies can be considered.

## Materials and Methods

### Primary neonatal rat ventricular cardiomyocyte isolation

All animal studies were approved by the University College London Biological Services Ethical Review Committee and licensed under the UK Home Office regulations and the Guidance for the Operation of Animals (Scientific Procedures) Act 1986 (Home Office, London, United Kingdom). Primary cardiomyocytes were isolated as previously described [44]. All isolation buffers were oxygenated by bubbling medical grade oxygen through the solution for 5 minutes prior to use. Neonate rat pups (Sprague-Dawley) that were <2 day postpartum were decapitated and rinsed in ethanol. The hearts were removed by cutting along the sternum and dissecting the heart through the chest wall. Hearts were washed in isolation buffer (116 mM NaCl, 20mM HEPES, 0.77 mM NaH_2_PO, 5.5 mM Glucose, 5.4 mM KCl, 0.4 mM MgSO containing 600 μg/ml collagenase type II and 250 μg/ml pancreatin) and cut into small 2 mm pieces and then incubated at 37°C until plating. The digestion procedure was repeated 7 times and the pellets pooled and pre-plated for 1 hour in 15% (v/v) Foetal Bovine Serum (FBS) in DMEM to remove contaminating fibroblasts. Cardiomyocytes were then seeded at 2 x 10^6^ cells per well of a 6 well plate, which had been pre-coated with 1% (w/v) gelatin (Sigma-Aldrich). The DMEM containing 15% (v/v) FBS was replace the following day with maintenance media (DMEM containing 1% (v/v) FBS) to ensure fibroblast contamination was restricted. Cell purity was assessed by immunofluorescence, with desmin a myocyte specific marker confirming that fibroblast contamination was minimal (data not shown).

### *In vitro* simulated I/R injury

Cell culture media was replaced with ischaemic buffer (137 mM NaCl, 12 mM KCl, 0.49 mM MgCl, 0.9 mM CaCl_2_.H_2_0, 4 mM HEPES, 20 mM sodium lactate and 10 mM deoxyglucose [pH 6.2]) and cells transferred to an in-house built ischaemic chamber pre-warmed to 37°C. Simulated ischaemia was achieved by addition of 5% CO_2_ in balanced argon to exclude any oxygen. Cells were subjected to simulated ischaemic injury for 4 h after which ischaemic buffer was replaced with DMEM containing 1% (v/v) FBS and cultured in 5% CO_2_ in air (simulated reperfusion) for the indicated times.

### *In vivo* cardiac I/R injury model

All studies were performed using Male Sprague Dawley (SD) rats (Charles River, UK) weighing between 200–220g, receiving a standard diet and water *ad libitum*. Rats were dosed with 200 mg/kg HCQ, administered daily by gavage, for three days prior to surgery (this did not alter rat weight or food and water intake). This achieved blood concentrations of 1000–2000 ng/ml which corresponds to therapeutic doses achieved in patients (Fig 5D). The ERK1/2 inhibitor, U0126, was administered via IP injection (1mg/kg), 30 minutes prior to surgery. The left anterior descending (LAD) coronary artery of male SD rats was occluded using methods previously described [45]. In brief, rats were anaesthetised using using 2% isoflurane in O_2_, and a thoracotomy was performed. The pericardium was removed and a 5–0 prolene suture placed under the LAD, about 2 mm from the origin. The suture was tied around a small piece of PE tubing, occluding the LAD and left for 1 hour. The tubing was then removed to allow reperfusion for 24 h. Rats were observed continuously during recovery for signs of distress or pain by trained personnel and in accordance with the universities guidelines. When rats were sacrificed they were anaesthetised using sodium pentobarbital (50 mg/kg). Their hearts were removed and stained with Evans blue and TTC to assess area of infarct size (IS) and area at risk (AAR) (Fig 5B and 5C). AAR was shown to be consistent confirming reproducibility of technique (Fig 5C).

### Whole blood hydroxychloroquine detection assay

HCQ concentrations in rat whole blood were assayed by using high performance liquid chromatography (HPLC) coupled with fluorescence detection. This assay was performed by Dr Benoit Blanchet, Laboratory of Pharmacology, Hôpitaux de Paris. Chromatographic separation was achieved on Xterra Phenyl (250 mm x 4.6 mm, 5 μm; Waters, Milford, USA) associated with a guard column packed with the same bonded phase. The mobile phase consisted of a mixture of glycine buffer (pH 9.6, 100 mM,) and methanol (46:54; v/v), and was delivered at a flow rate of 1.2 mL/min throughout the 16-minute run. Chromatography was performed at 50°C. The excitation and emission wavelengths are 320 and 380 nm, respectively.

Regarding sample preparation, 20 μl of quinine (internal standard) at 50 μg/mL was added to 200 μl of whole blood (calibration standard, internal quality control or rat sample). After mixing, 400 μl of cold methanol and 50 μl of cupric sulphate (3 nM) were added before a 2-minute vortex step with a VX-2500 Multi-Tube Vortexer (VWR, Fontenay Sous Bois, France). Then, the tubes were centrifuged 15 minutes (13000 rpm, room temperature). The supernatant was transferred into a plastic vial for chromatography, and then 20 μl of each sample was injected into the chromatographic system. Calibration curves were linear from 25 to 1560 ng/mL. The intra- and inter-assay coefficients of analytical variabilities were both less than 10%. The lower quantification limit was 25 ng/mL.

### Determination of apoptosis

Cells were seeded on UV irradiated coverslips and fixed in 4% (w/v) paraformaldehyde in phosphate buffered saline (PBS) for 15 min at room temperature. In situ cell death was detected in cultured cardiomyocytes by using Terminal Deoxynucleotidyl Transferase-Mediated dUTP Nick-End Labelling (TUNEL) assay kit (Roche Diagnostics, Meylan, France) according to the manufacturer’s instructions. Cells were then washed and cell nuclei counter stained with DAPI. Coverslips were mounted onto glass slides using a Zeiss Axioscope inverted fluorescence microscope (Zeiss).

### Western Blotting

Cells were lysed in 1x RIPA buffer (20 mM TRIS-HCl [pH 7.5], 150 mM NaCl, 1 mM EDTA, 1 mM EGTA, 1% (v/v) IGEPAL, 1% (w/v) deoxycholate, 250 mM sodium pyrophosphate and Complete-mini protease inhibitor cocktail (Roche)) and incubated on ice for 15 min. Lysates were spun at 13, 000 rpm for 5 min to pellet cell debris. The supernatant was transferred to a clean tube and assayed for protein using the BCA assay kit according to the manufacturer’s instructions (Pierce). Twenty micrograms total protein was run on denaturing PAGE gels in protein running buffer (25 mM TRIS-HCl, 192 mM Glycine, 0.1% [w/v] sodium dodecyl sulfate) and wet-transferred in transfer buffer (25 mM TRIS-HCl, 192 mM Glycine, 0.1% [w/v] sodium dodecyl sulfate containing 20% [v/v] methanol) to Hybond-C nitrocellulose membrane (GE Healthcare). Membranes were blocked for 60 min in 5% (w/v) non-fat dry milk in PBS for 1 h at room temperature before being incubated with primary antibody (Cell Signalling) overnight at 1/1000 dilution in 5% (w/v) non-fat dry milk in PBS at 4°C. The following day they were washed 3 times in PBS containing 0.1% (v/v) Tween20. Horseradish-peroxidase conjugated secondary antibodies (DAKO) were used at 1/5,000 dilution in 5% (w/v) non-fat dry milk for 1 h at room temperature and then washed 3 times in PBS containing 0.1% (v/v) Tween20. Bands were visualized using enhanced chemiluminescence (GE Healthcare).

### Survival assay

The viability of the cells was assessed using CellTiter 96 Aqueous One Solution assay kit (Promega), an advanced MTT assay. This assay is based on a formazan reaction and therefore detects metabolic active cells, meaning apoptotic and necrotic cell death is measured. Following simulated I/R injury 100 μL of the solution was added to each well for 1h at 37°C, 5% CO_2_ incubator and then absorbance was read on a TECAN GENios Microplate reader at 492 nm. The percentage survival of cells was defined as (absorbance in each well)/(absorbance in control well) x 100 (%).

### Statistical analysis

Results are presented as mean ±standard error mean, and are from a minimum of three independent experiments unless otherwise stated. We determined the statistical significance of the difference between experimental groups in instances of single comparisons by the two-tailed unpaired Student’s t-test of the means. For multiple means comparisons, one-way analysis of variance (ANOVA) followed by Tukey post hoc test was used to determine statistical significance.

### Study approval

All animal studies were approved by the University College London Biological Services Ethical Review Committee and licensed under the UK Home Office regulations and the Guidance for the Operation of Animals (Scientific Procedures) Act 1986 (Home Office, London, United Kingdom)

## Supporting Information

S1 Fig HCQ is most protective when present before, during and after simulated I/R injury. Cells were pre-treated overnight with 2 μg/mL HCQ at various times; 1-pretreatment overnight, 2-treatment during simulated ischaemia (4h hypoxia), 3-treatment during simulated reperfusion (4h reoxygenation). Levels of cleaved caspase-3 (a) and ERK1/2 phosphorylation (b) were assessed using western blot. Graphs show ±SEM of quantitative analysis from four independent experiments. Statistical analysis determined by 1 way ANOVA using post-hoc Tukey to compare all columns (** p<0.005)(a).
(TIF)

S2 Fig The autophagy inhibitor 3MA had no effect on cleavage of caspase-3 in neonatal rat cardiomyocytes in simulated cardiac I/R injury. Western blot analysis of cell lysates from neonatal rat cardiomyocytes pre-treated overnight with 2 μg/mL HCQ and the following day incubated with 5 μM 3MA for 2 hours followed by simulated I/R injury (4h hypoxia + 2h reoxygenation). Protein lysates were collected and immunoblots probed for LC3-II/LC3-I (a), ERK1/2 phosphorylation (b) and cleaved caspase-3). Graphs show mean ±SEM of quantitative analysis from five (a) and three (b+c) independent experiments. Statistical analysis determined by 1 way ANOVA using post hoc Tukey to compare all columns (*p<0.05)(a).
(TIF)
