# Supplementary figures and images for: Hydroxychloroquine Protects against Cardiac Ischaemia/Reperfusion Injury In Vivo via Enhancement of ERK1/2 Phosphorylation
Source: PLoS One. 2015 Dec 4;10(12):e0143771. doi: 10.1371/journal.pone.0143771 (PMC4670100; doi:10.1371/journal.pone.0143771)

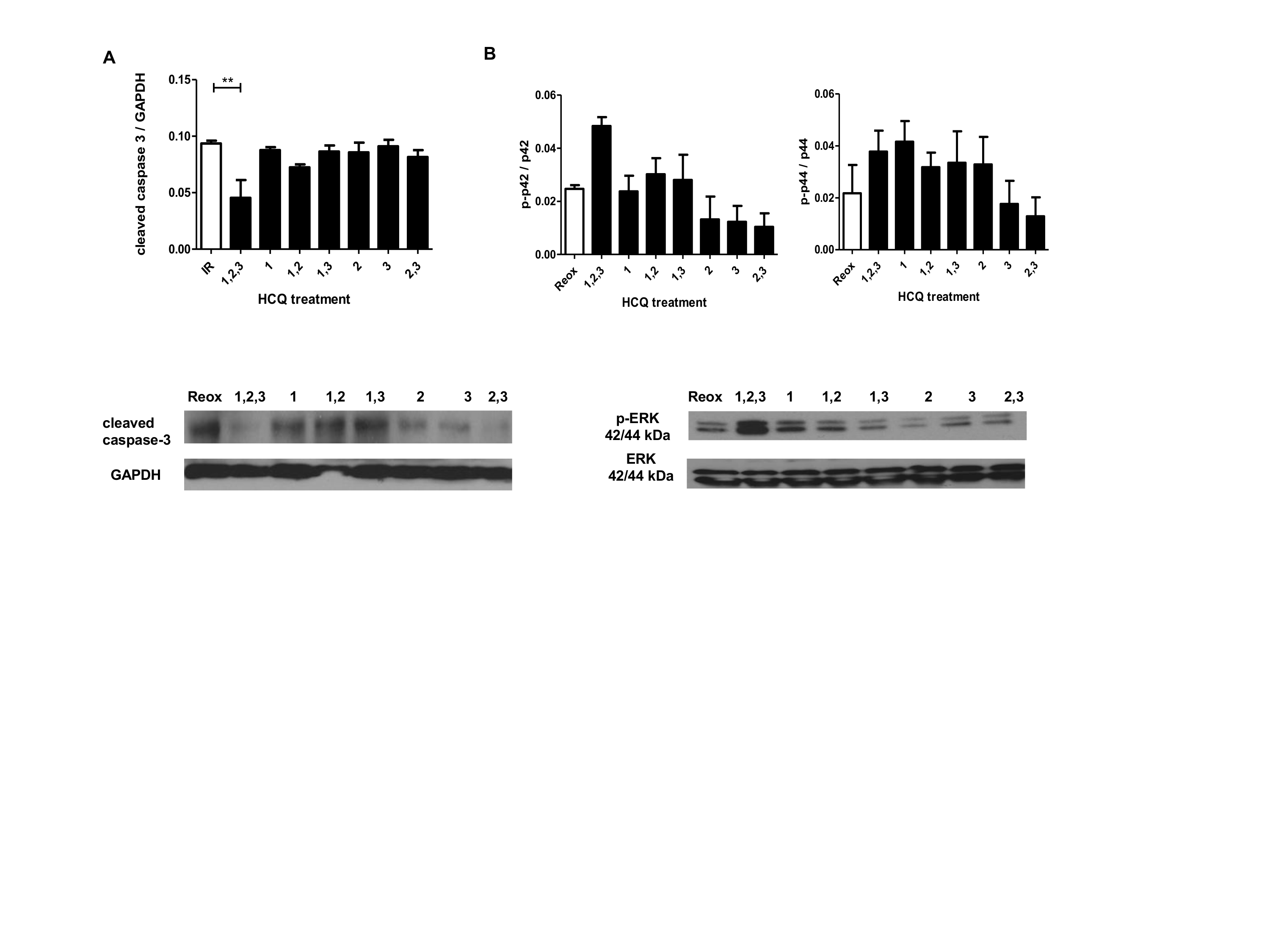

Supplement: S1 Fig — Cells were pre-treated overnight with 2 μg/mL HCQ at various times; 1-pretreatment overnight, 2-treatment during simulated ischaemia (4h hypoxia), 3-treatment during simulated reperfusion (4h reoxygenation). Levels of cleaved caspase-3 (a) and ERK1/2 phosphorylation (b) were assessed using western blot. Graphs show ±SEM of quantitative analysis from four independent experiments. Statistical analysis determined by 1 way ANOVA using post-hoc Tukey to compare all columns (** p<0.005)(a). (TIF) [file pone.0143771.s001.tif]

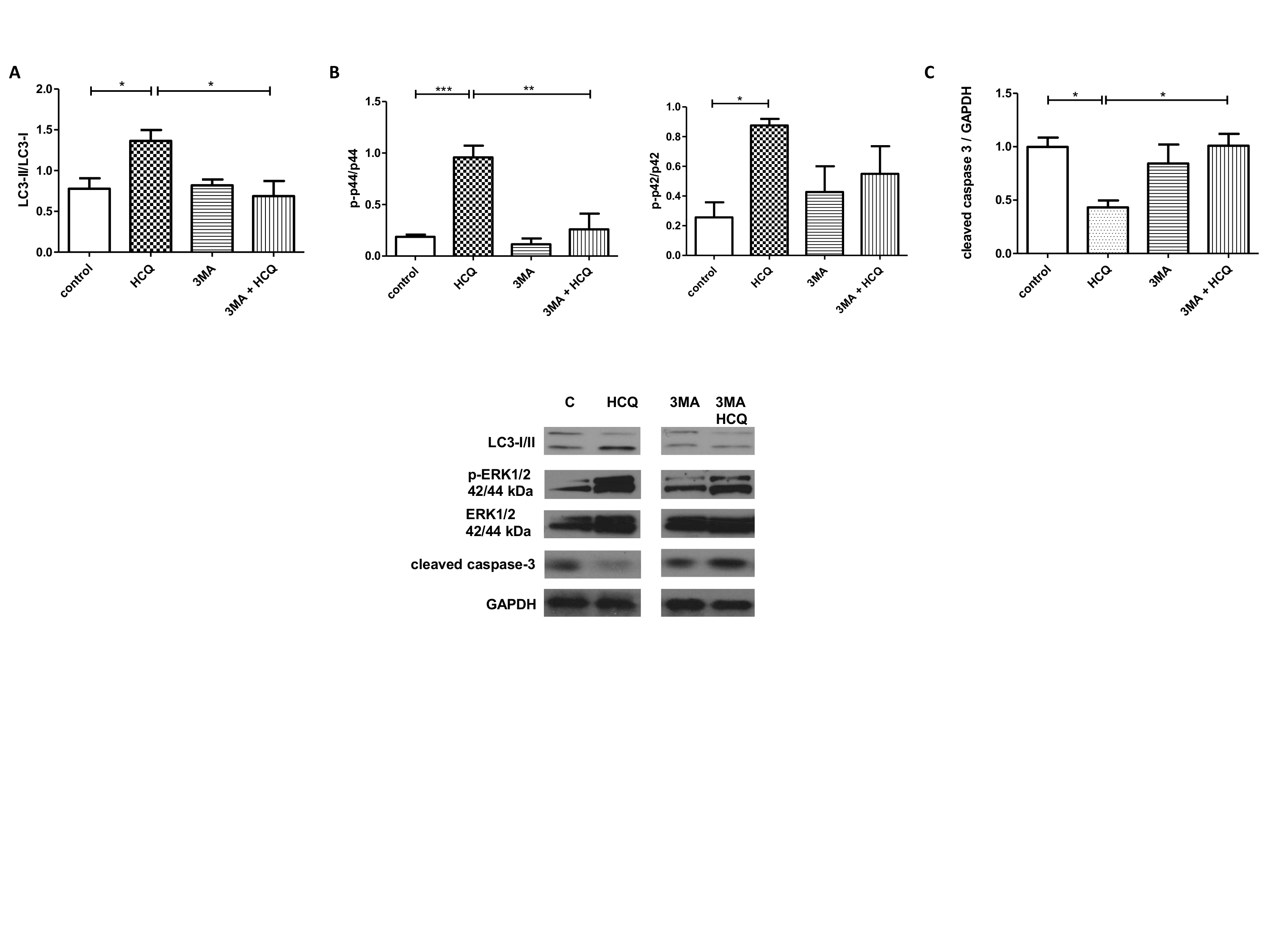

Supplement: S2 Fig — Western blot analysis of cell lysates from neonatal rat cardiomyocytes pre-treated overnight with 2 μg/mL HCQ and the following day incubated with 5 μM 3MA for 2 hours followed by simulated I/R injury (4h hypoxia + 2h reoxygenation). Protein lysates were collected and immunoblots probed for LC3-II/LC3-I (a), ERK1/2 phosphorylation (b) and cleaved caspase-3). Graphs show mean ±SEM of quantitative analysis from five (a) and three (b+c) independent experiments. Statistical analysis determined by 1 way ANOVA using post hoc Tukey to compare all columns (*p<0.05)(a). (TIF) [file pone.0143771.s002.tif]
